# Impact of Nanoparticle Additions on Life Cycle Assessment (LCA) of Ceramic Tiles Production

**DOI:** 10.3390/nano14110910

**Published:** 2024-05-22

**Authors:** Euler L. Saavedra, Johann F. Osma

**Affiliations:** 1 Doctoral Program in Management of Technological Innovation, School of Engineering, Universidad de los Andes, Cra. 1E No. 19a-40, Bogotá 111711, Colombia; e.saavedra@uniandes.edu.co; 2 Department of Electrical and Electronic Engineering, Universidad de los Andes, Cra. 1E No. 19a-40, Bogotá 111711, Colombia; 3 Department of Biomedical Engineering, Universidad de los Andes, Cra. 1E No. 19a-40, Bogotá 111711, Colombia

**Keywords:** ceramic tiles, energy savings, life cycle assessment (LCA), nanoparticles, sustainability, building materials

## Abstract

The ceramic tile industry, with significant energy and material demands in its manufacturing processes, has employed technological innovations in energy efficiency, advanced equipment and tile thickness reduction to address these challenges. This study aimed to assess the impact of Ag_2_O, CuFe_2_O_4_, Fe_3_O_4_, and SiO_2_ nanoparticles (0%, 1%, and 5% by weight) on the mechanical strength, water absorption, and apparent thermal conductivity of ceramic tiles, as well as their capacity to reduce energy and raw material consumption. This reduction translates into a decrease in environmental impacts, which have been evaluated through life cycle assessment (LCA) methodology applied to the manufacturing processes. Nanoparticles (Ag_2_O, CuFe_2_O_4_, Fe_3_O_4_, and SiO_2_) were initially screened on TF clay (0%, 1%, 5% *w*/*w*), and the most effective were applied to CR1 and CR2 clays (0%, 1%, 5% *w*/*w*). Findings indicated a 32% increase in temperature gradient and a 16% improvement in flexural strength with the addition of Fe_3_O_4_ nanoparticle at 1% (*w*/*w*) in TF clay. Furthermore, there was a potential 48% reduction in energy consumption, and up to 16% decrease in tile weight or thickness without affecting the flexural strength property of the test tiles. LCA results demonstrated that the addition of Fe_3_O_4_ nanoparticle has potential reductions of up to 20% in environmental impacts. This study suggests that nanoparticle addition offers a viable alternative for reducing energy and material consumption in the ceramic tile industry. Future research should focus on assessing the economic impact of transitioning to a sustainable business model in the ceramic tile industry with nanoparticles addition.

## 1. Introduction

Ceramic tile is one of the most widely used materials in building construction [1]. Ceramics are inorganic and non-metallic materials mainly used in walls, floors and ceilings, characterized by their high chemical and physical properties, such water absorption and flexural strength, as well as its high production and relevant economic value [2]. The primary components used in the production of standard ceramic bodies, such as monoporosa and porcelain stoneware tiles, are clay, feldspar, and quartz [3]. In 2022, world ceramic production was 16,762 million square meters, down 9.7% from 2021 [4]. The production of ceramic tiles involves types of energy sources such as electric and thermal. Electricity is used to generate mechanical force, and thermal energy, such as fuels, natural gas, liquefied petroleum gas and coal, is used as direct heat in the different drying and firing processes [1,5]. It is estimated that, in the ceramic process, approximately 8–12% of the total energy consumed is electrical and 88–92% is thermal [6,7]. A significant amount of thermal energy is used in the atomizing process (36%), drying (9%) and firing (55%) [8,9].

The study by Delpech et al. [10] found that thermal energy gives rise to air emissions of carbon dioxide, which are associated with gas combustion during the ceramic process. Now, these emissions are subject to control and reduction measures. For that reason, it is imperative to consider all the possible environmental and health impacts related to the ceramic process in order to improve the environmental sustainability of this process. Several authors have identified strategies to improve energy efficiency in the different processes of the ceramic industry, ranging from waste heat utilization [11,12,13,14], equipment optimization [8] and incorporation of renewable energy use in the atomizing [15,16,17,18], drying and firing processes. Few studies have focused on the reduction of thickness to improve energy efficiency and the use of materials in other ceramic tile groups such as porcelains tiles [9,19,20,21], whose apparent thermal conductivity has not been considered as an important characteristic for the reduction of energy consumption.

On the other hand, ceramic tile manufacturing companies have developed new technologies and products in recent years [22]. Some of these approaches have focused on finding new functions of their surfaces, with the aim of offering some new attributes beyond the conventional ones [23]. The new attributes or functionalities are aimed at improving aspects of tile production related to the quality of life of people, uses of ceramic tiles in new spaces and in the production of solar energy [24,25,26,27].

Several studies and patented researches have been carried out that have incorporated various types of nanoparticles, such as SiO_2_, Ge, TiO_2_, Cu, Au, Ag, Pt, Ti and Mg with the aim of improving the photocatalytic, antibacterial and self-cleaning properties of coating glazes [28,29,30,31,32,33,34,35,36,37,38,39], as well as enhancing the flexural strength of ceramic tile bodies through the addition of SiO_2_, PbO, Al_2_O_3_, Fe_2_O_3_, Ca, Na; Mg; B, Zn, Al and P nanoparticles [40,41,42,43,44].

In the study conducted by Chen et al. [40], replacements of different proportions of potter’s clay and porcelain clay with incinerated sludge ash (ISSA) were carried out to fabricate sludge ash tile samples. These tiles were used to examine the effect of incorporating nano-SiO_2_ particles (average particle size of 10 ± 5 nm) as strengthening additives in the clay-ISSA materials. The increase in flexural strength was 0–2.5 MPa.

The effects of the incorporation of nano-alumina (Al_2_O_3_) and nano-silica (SiO_2_) in porcelain stoneware tiles were evaluated by De la Garza et al. [42]. The tiles were manufactured with concentrations of 0.25, 0.5, 0.75 and 1 wt.% of Al_2_O_3_ and SiO_2_ nanoparticles with an average particle size between 20–30 nm. In this study, the authors evaluated key physic-mechanical properties, such as compressive strength, water absorption, density and porosity. The sample with 1 wt.% nano-SiO_2_ showed the highest compressive strength (65.27 MPa) related to the sample with the lowest porosity.

Fierro et al. [44] patented a process for the preparation, conditioning and stabilization of a family of basic additives sodium, potassium, boron, silicon, zinc, and calcium oxides, among others, prepared by physicochemical and chemical synthesis methods, forming nanometric structures, reformulated with deflocculants, sequestrants and dispersants additives, which allow one to obtain a dispersion or powder capable of reducing the sintering temperature of a ceramic body up to 13% due to the high fluxing power maximized by the use of nanotechnology in the structures obtained.

Mahmoud et al. [41] studied the incorporation of lead oxide (PbO) nanoparticles (mean particle size equal to 29.64 nm) into standard ceramic tiles, with concentrations from 0.0 to 10.0 wt.%, to serve as promising alternative candidates for γ-ray shielding. The modulus of rupture increased from 1.753 to 2.674 MPa.

Nawaukkaratharnant et al. [43] studied the addition of iron oxide particles to improve the properties of red stoneware tiles. The water absorption and apparent porosity of the samples decreased with increasing Fe_2_O_3_ content and firing temperature. Furthermore, the addition of Fe_2_O_3_ up to a total content of 8.05 wt.% improved the flexural strength up to 30 MPa and reduced the firing temperature; also, the water absorption was reduced to less than 1.5% after firing at 1150 °C. In contrast, the flexural strength was reduced with a total Fe_2_O_3_ content of up to 9.99 wt.%.

The ceramic industry is currently facing significant environmental challenges, and the approach of improving ceramic properties through the addition of nanoparticles is relevant. An LCA study allows us to quantify the various impacts of the cradle-to-gate ceramic tile manufacturing process. This means that it allows us to evaluate the impact of this process and is not necessarily related to the toxicity of the final product. Therefore, it is important to quantify the impact of the process to assess whether the incorporation of nanoparticles has a positive effect in reducing the impact on the production of ceramic tiles. Numerous studies have highlighted the importance of life cycle assessment (LCA) as a fundamental tool for addressing environmental impacts on industrial ceramic sector in order to identify opportunities for improving sustainability [45,46,47,48,49,50,51,52,53]. According to recent studies in different countries, such as the one conducted by Boschi et al. [2] in Italy that evaluated environmental impacts in Italian tile production, reductions in the environmental footprint across 90 factories were shown. The study carried out by Wang et al. [5] in China quantified environmental improvements in ceramic processes, comparing traditional and cleaner production technologies. Another study by Ros-Dosda et al. [9] in Spain examined the environmental impacts in the life cycle of porcelain stoneware tiles, focusing on thickness reduction and glazing usage. Meanwhile, Türkmen et al. [54] in Turkey compared impacts of current and cleaner manufacturing scenarios, considering factors like drying heat recovery and thickness reduction in a “cradle to gate” approach.

Although nanoparticles have found applications in ceramic tiles, studies and patents are primarily focused on enhancing the photocatalytic, antibacterial, and self-cleaning properties of coatings [28,29,30,31,32,33,34,35,36,37,38,39]. However, some research on ceramic tiles has explored alternatives to improve the mechanical properties of the ceramic body, such as flexural strength by adding nanoparticles of Al_2_O_3_, SiO_2_, PbO, and Fe_2_O_3_ [40,41,42,43,44]. In our study, we have incorporated SiO_2_ nanoparticles because of their widespread use in previous studies [40,42]. Additionally, three types of nanoparticles (Ag_2_O, CuFe_2_O_4_, and Fe_3_O_4_) were included owing to their excellent apparent thermal conductivity and limited utilization in prior studies. These nanoparticles not only enhance properties such as flexural strength and apparent thermal conductivity of ceramic tiles, but also have the potential to reduce energy and raw material consumption. This reduction translates into decreasing environmental impacts, evaluated through life cycle assessment (LCA) methodology applied to the manufacturing processes. The aim of this study is to evaluate how the addition of Ag_2_O, CuFe_2_O_4_, Fe_3_O_4_ and SiO_2_ nanoparticles in three different concentrations (0%, 1%, 5% by weight) improves properties such as flexural strength and apparent thermal conductivity in test tiles made from three types of clays (CR1, CR2 and TF). Initially, a screening of nanoparticles was conducted using TF clay, followed by testing the most promising ones in terms of improving the properties of ceramic tiles with CR1 and CR2 clays.

## 2. Materials and Methods

### 2.1. Materials

Three red clays were used: CR1, CR2 and TF from Colombia (particles size, 12.7 mm > 22.0% > 7.9 mm; 7.9 mm > 4.6% > 6.4 mm; 6.4 mm > 7.8% > 4.0 mm; 4.0 mm > 33.6%> 1.0 mm; 1.0 mm < 32.0%). We used silver oxide (Ag_2_O) nanoparticles (particle size < 15 nm) from Smalticeram (Castellon, Spain), copper iron oxide (CuFe_2_O_4_) nanoparticles (particle size < 100 nm) from Sigma Aldrich (Taufkirchen, Germany), magnetite (Fe_3_O_4_) nanoparticles (particle size < 88.59 nm) from the Ceramica Italia laboratory (Cucuta, Colombia), silicon dioxide (SiO_2_) nanoparticles (particle size < 20 nm) from Sigma Aldrich (Taufkirchen, Germany), and treated water. The chemical compositions of clays were examined by X-ray fluorescence spectroscopy; the results are shown in Table 1.

### 2.2. Equipment

Equipment used includes: magnetic stirrer MR Hei-Tec HEIDOLPH (Schwabach, Germany); measurement apparatus, Ceramica Italia (Cucuta, Colombia); K-type thermocouples, Instruequipos Ingenierias S.A.S. (Medellin, Colombia); 100 mL beaker, Brixco (Montevideo, Uruguay); four output data logging thermometers, model DT4947SD, General Tools & Instruments (New York, NY, USA); Chronometer HS 70, Casio (Tokyo, Japan); a plastic container (obtained from the local market); Grinder ct-12061, Servitech (Tubarao, Brazil); drying oven DVS600, Yamato Scientific Co., Ltd. (Tokyo, Japan); Grinder Speedy 1, Nannetti (Faenza, Italy); Oven N 20/HR, Nabertherm (Lilienthal, Germany); manual press type P.I.L.A, Sacmi (Imola, Italy); drying oven WTC, Binder (Tuttlingen, Germany); viscometer FC/100/A, Ceramics Instruments (Sassuolo, Italy); pycnometer GT0151, Gabrielli Technology (Calenzano, Italy); weighing machine GF-10002A, A&D Company (Tokyo, Japan); Moisture analyzer MX-50, A&D Company (Tokyo, Japan); grinding container GT0535, Gabrielli Technology (Calenzano, Italy); vernier caliper 530-104BR, Mitutoyo (Suzano, Brazil); sieve GT0452 ASTM No. 18 [55], Gabrielli Technology (Calenzano, Italy); sieve GT0463 ASTM No. 230 [55], Gabrielli Technology (Calenzano, Italy); strength tester CR4-650, Gabrielli Technology (Calenzano, Italy); a metal container, 21 cm × 17 cm (obtained from the local market); and a metal tray, 32 cm × 43 cm × 5 cm (obtained from the local market).

### 2.3. Test Tiles Manufacturing Process

**Figure 1:**
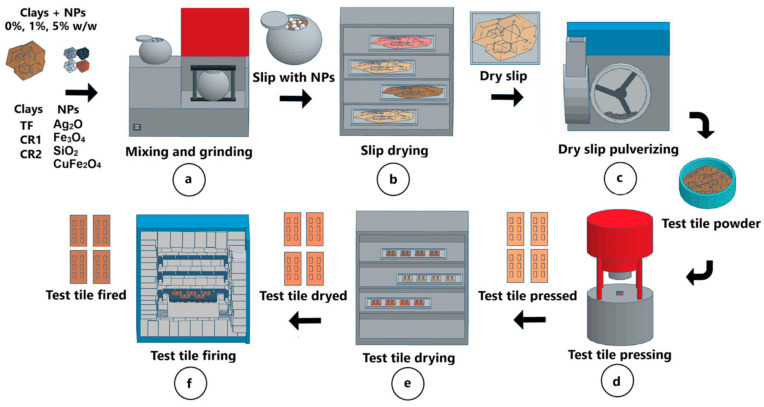
Ceramic test tiles manufacturing process.

The general manufacturing process for the sample tiles is shown in Figure 1. The mixing and milling process started with the preparation of 500 g of dry sample mass (Dsm) of each clay TF, CR1 and CR2 Figure 1a. Initial moisture percentages (%M) were determined for each of the clay samples on the MX-50 moisture analyzer (A&D Company Tokyo, Japan). The wet sample mass (*Wsm*) for each material was calculated using Equation (1) [56]:

(1) $$Wsm=\frac{Dsm}{100-M}\times 100\%$$

The amount of water for the grinding process was calculated based on the relation of 65% solids (S) and 35% water (W) by Equation (2) [57]:

(2) $$Amount\;of\;water=\frac{\left(Dsm\times W\right)}{S}$$

Also, the net amount of water to be added to the sample was calculated using Equation (3) [57]:

(3) Net water added=Amount of water−Wsm

Then, 750 g of alumina balls in three different sizes were loaded into grinder container GT0535. The first size was about 10–14 mm in diameter, corresponding to 15% of the load weight, the second size was 16–19 mm in diameter, corresponding to 35% of the load weight, and finally, the third size was 20–25 mm, corresponding to 50% of the load. For the 0% (*w*/*w*) concentration of nanoparticles, each of the clays was ground separately; the 500 g dry base, the net amount of water and additives (deflocculant) were added to the GT0535 vessel and then ground in the Speedy 1 for 5 min to obtain the slip. In the case of grinding clays with 1% and 5% (*w*/*w*) concentration of nanoparticles (Ag_2_O, CuFe_2_O_4_, Fe_3_O_4_ and SiO_2_), the dry basis reference was the same, i.e., the sum of the dry mass of clay and nanoparticles was 500 g. The density (g/cm^3^) and viscosity (g/cm·s) conditions were measured in the GT0151 pycnometer and FC/100/A viscometer, respectively. The slip was deposited on the metal tray, and drying was carried out in DVS600 at a temperature between 150 °C and 170 °C for 120 min (Figure 1b). When the dry sample was obtained, it was ground in CT-12061, moistened according to the plasticity index (5.6%), sieved in GT0452, and reposed for 20 min Figure 1c.

For the pressing process of the test tile (Figure 1d), 85 g of material were weighed for each of the test pieces at a pressure of 240 Bar in the manual press P.I.L.A. type. Four rectangular test tiles of dimensions 100.5 mm in length, 50 mm in width and thickness of 7.5 mm were obtained by each nanoparticle clay mixture (0%, 1% and 5% *w*/*w*). Then, each test tile was dried at 140 (±5) °C in oven WTC for 120 min, obtaining a humidity of less than 1% by weight (Figure 1e). After drying, each test tile was measured for length (L dry), width (A dry), and weight (Wdry) with the Vernier caliper 530-104BR and the GF-10002A weighing machine.

**Figure 2:**
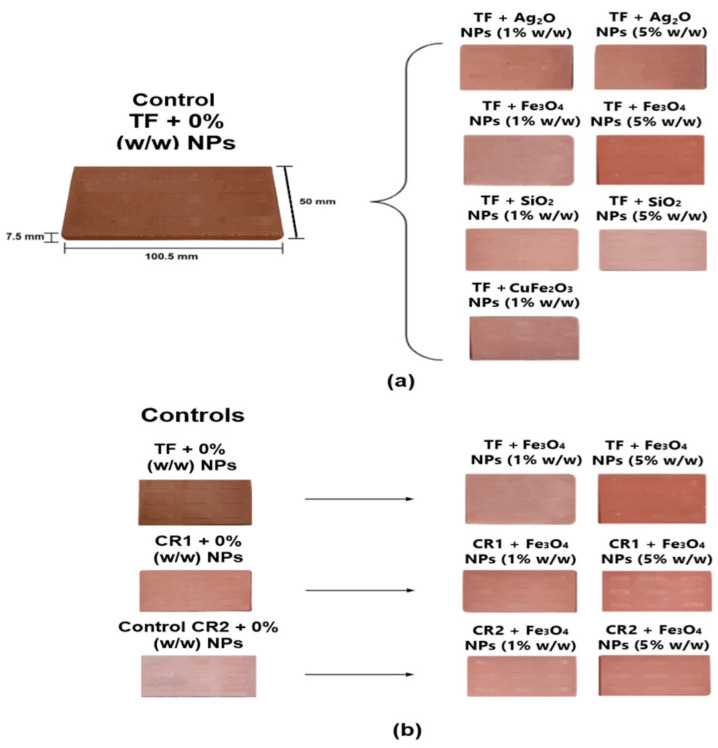
Clay test tiles with nanoparticles addition fired at 1120 °C. (**a**) TF control clay test tiles with 0%, 1% and 5% (*w*/*w*) additions of Ag_2_O, CuFe_2_O_4_, Fe_3_O_4_ and SiO_2_ nanoparticles. (**b**) Control clay test tiles for TF, CR1 and CR2 with additions of 0%, 1% and 5% (*w*/*w*) of Fe_3_O_4_ nanoparticles.

The automatic firing cycle in the N 20/HR oven was 37 min: 560 °C for 9 min, 570 °C for 3 min, 1120 °C for 4 min, 1120 °C for 3 min, 570 °C for 3 min and 560 °C for 15 min. Then, the kiln temperature gradually decreased to 100 °C, and then down to room temperature during a period of 24 h Figure 1f. Then, measurements of length (L fired), width (A fired) and weight (W fired) were obtained with the vernier caliper 530-104BR and the GF-10002A weighing machine. Figure 2 shows all types of test tiles of clays TF, CR1 and CR2 fired at 1120 °C. All tiles manufactured in this study follow the methodology described in this section.

### 2.4. Method for Calculating Percentage Linear Shrinkage (%LS), Loss on Fire (%LOF), Water Absorption (%WA) and Flexural Strength

We obtained the dimensional measurements for each dry (*L0*) and fired (*L1*) test tile with the 530-104BR equipment. Each sample represents three replicates. The firing linear shrinkage (*LS%)* was calculated for all test tiles using Equation (4) [42,57]:

(4) $$LS=\frac{L0-L1}{L0}\times 100\%$$

The fired test tile (*W*_1_) and dry test tile (*W*_0_) were weighed on GF-10002A. The loss on fire (*LOF%*) was calculated for all test tiles using Equation (5) [57,58]:

(5) $$LOF=\frac{W1-W0}{W1}\times 100\%$$

The percentage of water absorption (%WA) was determined according to the ISO 10545-3 standard [59]. The flexural strength was made in three replicates to determine the value of the flexural strength (N) test according to the ISO 10545-4 standard [60] on strength tester CR4-650.

### 2.5. Apparent Thermal Conductivity Test

**Figure 3:**
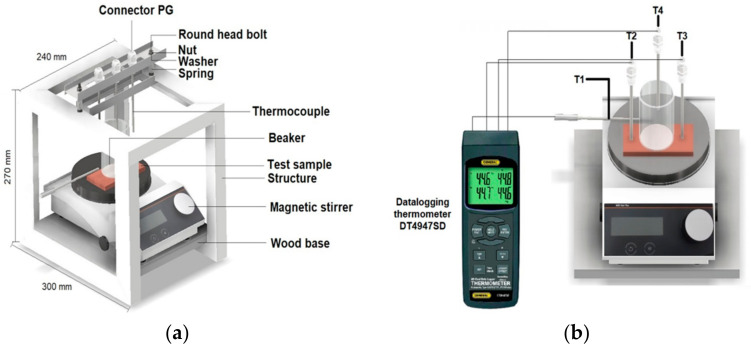
Apparent thermal conductivity test: (**a**) set up, (**b**) real measurement.

The thermal conductivity of a material represents the heat flow per unit thickness through a surface area, driven by a temperature gradient of one unit, while adhering to specific conditions [61,62,63]. The operating technique was based on the principle of heat transfer by steady-state conduction under laboratory conditions between the test tile at room temperature and a hot plate (Figure 3). For the measurement and storage of temperature data, it used the data-logging thermometer DT4947SD and 4 K-type thermocouples to obtain the temperature measurements.

In the apparatus assembly, the thermocouple 1 is connected to the magnetic stirrer plate surface, thermocouples 2 and 3 are positioned on the right and left side of the test sample, respectively, and thermocouple 4 is put inside the beaker with water. It was verified that the thermocouples were correctly connected to the data-logging thermometer DT4947SD. For the measurement of the temperature gradient, the temperatures of the thermocouples T1 (temperature of the magnetic stirrer MR Hei-Tec) and T4 (temperature of the water in the beaker) were taken as references in order to determine the delta of temperature (ΔT), which indicates the amount of heat transferred by the piece to the beaker with water. Once the stirrer was turned on and its set point was set at 100 °C, the beaker was filled with 100 mL of treated water. The data-logging thermometer was turned on, and the K-type thermocouples T1, T2, T3 and T4 were connected, then the test started.

The test sample was placed on the magnetic stirrer as centered as possible for uniform heat absorption, thermocouple T1 was placed on the stirrer plate, thermocouples T2 and T3 were lowered to make contact with the test sample, and thermocouple T4 was placed inside the beaker filled with water. Each sample represents three replicates. Data-logging was started and set to capture the data every 10 s for 15 min (*t* time). Then, the data-logging thermometer recording was stopped, and the beaker and thermocouples were removed. The data was exported to an Excel database, where the equation of the line was calculated for T4 (*Ti* initial and *Tf* final temperature) to obtain the slope (temperature gradient) and measure the increase of the water temperature. The temperature gradient was calculated by Equation (6).

(6) $$Temperature\;gradient\;\left(\frac{℃}{min}\right)=\left(Ti-Tf\right)/t$$

### 2.6. Characterization of Clays

Three clays (TF, CR1 and CR2) were tested with used clays in the manufacturing process of monoporosa cerasmic tile bodies in Colombia. A physical-ceramic characterization of their linear shrinkage (%), fire losses (%), water absorption (%) and flexural strength (Newtons) and temperature gradient (°C/min) properties was determined according to ISO 13006 [21] and ISO 10545-4 [60]. The temperature gradient (°C/min) was determined by respective test. The chemical compositions of clays were examined by X-ray fluorescence spectroscopy.

### 2.7. Study of Nanoparticles Selection

TF clay was used as a model for screening nanoparticles of Ag_2_O, CuFe_2_O_4_, Fe_3_O_4_ and SiO_2_. Mixtures (*w*/*w*) of TF with nanoparticles at 0%, 1% and 5% were tested. Variables of water absorption, temperature gradient and flexural strength were measured on each test tile. A physical-ceramic characterization of their linear shrinkage (%), fire losses (%), water absorption (%) and flexural strength (Newtons) properties was determined according to ISO 13006 and ISO 10545-4. The temperature gradient (°C/min) was determined by the respective test.

### 2.8. Study of Clays with Nanoparticles Addition

Nanoparticles with the best apparent thermal conductivity and flexural strength properties were also used, in addition at 0%, 1% and 5% (*w*/*w*) with CR1 and CR2 clays, to evaluate the effectiveness in linear shrinkage (%), fire losses (%), water absorption (%) and flexural strength (N) properties, determined according to ISO 13006 and ISO 10545-4, in these clays. The temperature gradient (°C/min) was determined by respective test.

### 2.9. Microstructural and Morphological Analysis TF Test Tile with Fe_3_O_4_ Nanoparticles Addition

The microstructure of the test tile fired was investigated using scanning electron microscopy (SEM) equipped with an energy dispersive X-ray spectroscopy detector (Coxem-CX200). To analyze the total percentage of porosity in TF test tile with Fe_3_O_4_ nanoparticles at 0%, 1% and 5% (*w*/*w*). Image processing software (ImageJ, version 1.54) was utilized. The total porosity percentage was calculated by comparing the pore area to the total area of the SEM image [43,44]. Three images were analyzed for each formulation.

### 2.10. Life Cycle Assessment

The LCA study, conducted in accordance with ISO 14040 guidelines [64], aimed to comprehensively evaluate the potential environmental impacts of ceramic tile production processes, specifically focusing on the use of CR1, CR2 and TF clays with magnetite (Fe_3_O_4_) nanoparticle additions at 0% and 1% (*w*/*w*). This assessment was based on an attributional “cradle to gate” approach encompassing laboratory-scale processes.

The life cycle impact assessment was carried out with Open LCA® software V1.11.0, using the Ecoinvent 3.8 database. The selection of impact categories was the ReCiPe 2016 midpoint (H) method, considering eighteen impact categories: fine particulate matter formation (FPMF), fossil resource scarcity (FRS), freshwater ecotoxicity (FE), freshwater eutrophication (FEP), global warming (GW), human carcinogenic toxicity (HCT), human non-carcinogenic toxicity (HNCT), ionizing radiation (IR), land use (LU), marine ecotoxicity (ME), marine eutrophication (MEP) mineral resource scarcity (MRS), ozone formation, human health (OF-HH), ozone formation, terrestrial ecosystem (OF-TE), stratospheric ozone depletion (SOD), terrestrial acidification (TA), terrestrial ecotoxicity (TE), and water consumption (WC).

**Figure 4:**
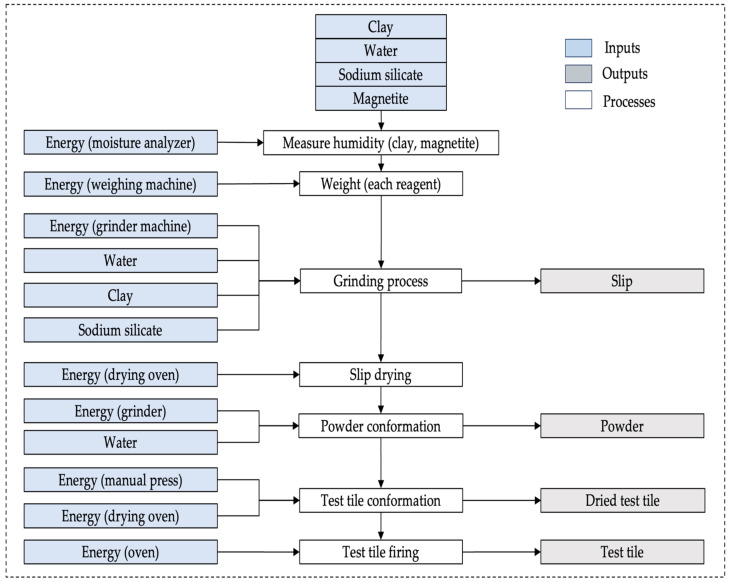
System boundaries of test tile manufacturing with magnetite addition.

Figure 4 shows the system boundaries were set from the use of raw materials until the obtaining of the sample tiles. It only considered the materials, water and energy consumption in the ceramic process production and magnetite synthesis manufacturing. The functional unit for the ceramic process was 420 g of material, and the functional unit for the nanoparticles was 5 g. The general unit of the process was the test tile with clays and nanoparticles.

### 2.11. Life Cycle Inventory (LCI)

The inventory of the relevant flows in the use of reagents and energy processes was carried out for the LCA study. Data on the input of raw materials, water and energy used in the ceramic process were captured in situ. Table 2 shows the inventory report for each manufacturing process of test samples, raw materials, measurement equipment, water consumption and energy required for the process. The clay type incorporated in the inventory report was generic for TF, CR1 and CR2 with the magnetite nanoparticles addition at 0% and 1% (*w*/*w*).

## 3. Results

### 3.1. Characterization of Clays

Chemical analyses of CR1, CR2 and TF clays by XRF (see Table 1.) reveal a high content of silicon (SiO_2_) and aluminum (Al_2_O_3_), which represent more than 80% of the total content. The presence of iron was evidenced by the red color of the sample tiles after firing [3]. All three clays contain iron oxide (Fe_2_O_3_); these do not exceed 4.4% by weight. Consequently, these samples are considered silicoaluminous clays, in line with the presence of silicates according to X-ray diffraction (Table 3).

**Figure 5:**
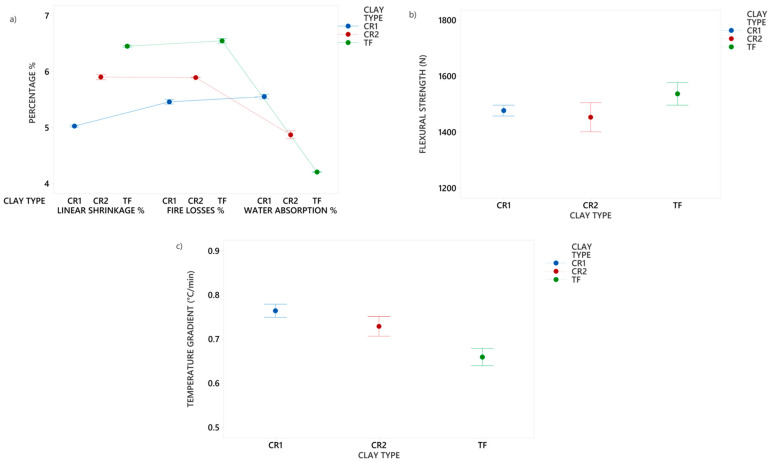
Interval plot of (**a**) linear shrinkage (%), fire losses (%), water absorption (%), (**b**) flexural strength (N) and (**c**) temperature gradient (°C/min) of CR1, CR2 and TF.

The test results for the ceramic characterization of CR1, CR2 and TF clays have been consolidated in Figure 5. The linear shrinkage is associated with the progressive sintering effect up to 1120 °C; greater shrinkage also results in increased mechanical properties [42]. Therefore, TF clay showed the highest percentage up to 6.5%. The fires losses of test tiles are associated with the elimination of organic matter and dihydroxylation of clay minerals (kaolinite) in the samples. The clay with the highest percentage of kaolinite (45%) was TF; consequently, it obtained 6.6% of fire losses.

Water absorption is an indicator of the densification and mechanical properties of ceramic tiles, based on the number of open pores accessible to water within the fired sample and the relationships with linear shrinkage follow an inverse correlation. In addition, it was observed that the relationships between water absorption and linear shrinkage have an inverse correlation [65]. The TF clay was the test tile with the lowest percentage of water absorption (4.2%) compared with CR1 and CR2 clays. All clays exceeded the 800 N of flexural strength established by the ISO 13006 standard. TF clay represents the highest value in flexural strength (1591 N). In comparison to the other clays, TF clay demonstrated a 14% increase in flexural strength relative to CR1 clay and a 9% increase compared to CR2 clay. This notable increase in flexural strength in TF clay is attributed to its high linear shrinkage (6.5%) and low water absorption (4.2%). Therefore, TF clay has a higher level of sintering at 1120 °C [66].

Regarding the temperature gradient, CR1 and CR2 clays recorded the highest values, at 0.74 and 0.73 °C/min, respectively (see Figure 5c). In contrast, TF clay exhibited the lowest temperature gradient, at 0.66 °C/min. This suggests that TF clay underwent a smaller temperature fluctuation during the test in comparison to the other clays [58]. Some studies report that the increase in thermal conductivity is associated with high density, lower water absorption and high flexural strength [67,68,69,70].

### 3.2. Study of Nanoparticles Selection

**Figure 6:**
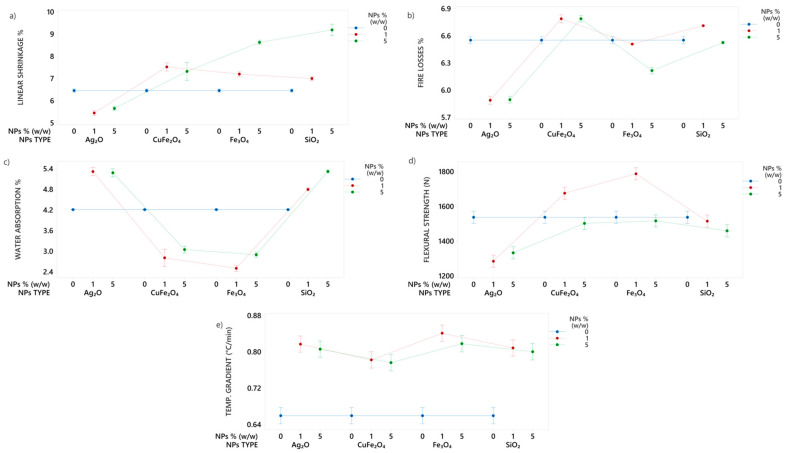
Interval plot of (**a**) linear shrinkage (%), (**b**) fire losses (%), (**c**) water absorption (%), (**d**) flexural strength (N) and (**e**) temperature gradient (°C/min) in TF clay with Ag_2_O, CuFe_2_O_4_, Fe_3_O_4_ and SiO_2_ nanoparticle additions.

Four types of nanoparticles (Ag_2_O, CuFe_2_O_4_, Fe_3_O_4_ and SiO_2_) at three concentrations (0%, 1% and 5% *w*/*w*) were tested on TF clay to determine the best-performing one, as measured by the five variables shown in Figure 6.

Findings regarding linear shrinkage showed increases in the test tile mixtures at 1% and 5% (*w*/*w*) CuFe_2_O_4_ (14–15%), Fe_3_O_4_ (12–34%) and SiO_2_ (9–43%) NPs, and decreases for Ag_2_O NPs (15–12%) compared to the 0% (*w*/*w*) mixture. Fire losses (Figure 6b) decreased up to 10% with Ag_2_O, and up to 5% with Fe_3_O_4_ at 1% and 5% (*w*/*w*) nanoparticle additions, and showed an increase of fire losses with CuFe_2_O_4_ and SiO_2_ nanoparticle additions, up to 3% and 2% nanoparticles additions, respectively. For water absorption (Figure 6c), Ag_2_O nanoparticles caused an increase up to 26% and 25% at 1% and 5% (*w*/*w*) to the 0% (*w*/*w*) mixture. This increase is caused by the appearance of a liquid phase that facilities porosity closure [71]. The addition of CuFe_2_O_4_ nanoparticles resulted in a decrease in water absorption of up to 33% and 26% at 1% and 5% (*w*/*w*), respectively. The addition of Fe_3_O_4_ nanoparticles caused a significant decrease in water absorption of up to 40% and 30% at 1% and 5% (*w*/*w*), respectively, compared to the 0% (*w*/*w*) mixture. In contrast, the addition of SiO_2_ nanoparticles resulted in an increase in water absorption of up to 14% and 28% at 1% and 5% (*w*/*w*), respectively, compared to the 0% (*w*/*w*) mixture. Finally, it is positive to find a low water absorption percentage because it means an adequate sintering rate or low porosity, and results in a higher flexural strength on ceramic tiles [54,66,72].

Figure 6d illustrates the flexural strength of each TF clay test tile. Only the TF clay test tiles with CuFe_2_O_4_ and Fe_3_O_4_ nanoparticle additions showed an increase in flexural strength up to 9% and 16%, respectively, at 1% (*w*/*w*) compared with the 0% (*w*/*w*) mixture. The low water absorption (2.8% and 2.5%) at 1% (*w*/*w*) magnetite nanoparticles indicate a higher density of the test tile and, consequently, a higher flexural strength (1787 N) promoted by the formation of a crystalline and liquid-phase developed at low-temperature associated with the iron oxide content [73,74,75]. Test tiles with the addition of Ag_2_O and SiO_2_ nanoparticles decreased the flexural strength by 13–16% and 1–5%, respectively, at 1% and 5% (*w*/*w*), compared at 0% mixture. The decrease in flexural strength can be due to reduction in the anorthite content generated by Ag_2_O and SiO_2_ nanoparticle additions [76]. CuFe_2_O_4_ and Fe_3_O_4_ nanoparticles showed a decrease in flexural strength by 2% and 1%, at 5% (*w*/*w*) with respect to the 0% (*w*/*w*) mixture. One of the causes is that the high iron oxide content generated an excess of liquid phase, which deteriorated the properties and the formation of high porosity structures [77,78]. Flexural strength results of the test tiles exceeded the value established (800 N) by ISO 13006 standard for ceramic tiles of group BIIb.

The temperature gradient from each TF clay test tile is illustrated in Figure 6e. All mixtures with 1% and 5% nanoparticles showed an increase in the temperature gradient with respect to the mixture with 0% nanoparticles addition. Test tiles with the addition of Ag_2_O and SiO_2_ nanoparticles at 1% and 5% (*w*/*w*) demonstrated a similar increase of temperature gradient of up to 24% and 21%, respectively. Test tiles with the addition of CuFe_2_O_4_ nanoparticles showed an increase of temperature gradient up to 18%. The temperature gradient increase for the test tiles with the addition of 1% and 5% (*w*/*w*) Fe_3_O_4_ nanoparticles was 32% and 28%, respectively. This increase is due to the decrease in the percentage of water absorption, which determines the density of test tiles. The results suggest that the addition of nanoparticles can improve the thermal properties of TF clay. The iron oxide content contributes to the formation of the glassy phase and favors densification, leading to an increase in thermal conductivity [27,79].

According to the study, magnetite (Fe_3_O_4_) nanoparticles at 1% (*w*/*w*) evidenced the highest increase in flexural strength (up to 16%) and temperature gradient (32%). From the results obtained, the magnetite (Fe_3_O_4_) nanoparticles were chosen to be tested with CR1 and CR2 clays.

### 3.3. Study of Clays with Nanoparticles Addition

**Figure 7:**
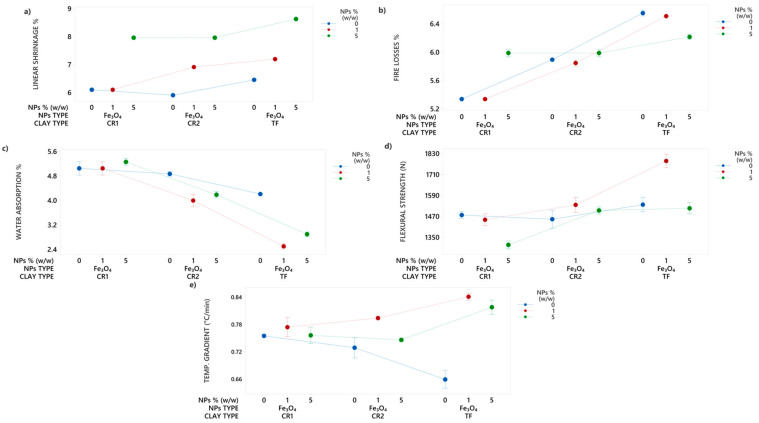
Interval plot of (**a**) linear shrinkage (%), (**b**) fire losses (%), (**c**) water absorption (%), (**d**) flexural strength (N) and (**e**) temperature gradient (°C/min) in CR1, CR2 and TF clay with Fe_3_O_4_ nanoparticle additions.

From the results obtained previously Fe_3_O_4_ nanoparticles were chosen to be tested with CR1 and CR2 clays. Figure 7 illustrates the ceramic properties. The linear shrinkage showed increases with Fe_3_O_4_ nanoparticles addition at 5% (*w*/*w*), in CR1 up to 31%, CR2 up to 35% and TF up to 34%, compared to the 0% (*w*/*w*) mixture Figure 7a. In fire losses (Figure 7b), the 1% magnetite mixtures in the clays remained the same, while in the 5% mixtures increased up to 12% in CR1, 2% in CR2 and decreased up to 5% in TF compared to the 0% nanoparticles mixture. Figure 7c, Fe_3_O_4_ nanoparticles caused a decrease in water absorption of up to 11% and 6% at 1% and 5% (*w*/*w*), respectively, in CR1 clay compared to the 0% (*w*/*w*) mixture. For CR2 clay, the addition of Fe_3_O_4_ nanoparticles resulted in a decrease in water absorption of up to 13% and 11% at 1% and 5% (*w*/*w*), respectively, compared to the 0% (*w*/*w*) mixture. These findings suggest that the addition of Fe_3_O_4_ nanoparticles at specific concentrations can significantly affect the water absorption of clay materials.

For CR1 clay, Figure 7d shows the addition of Fe_3_O_4_ nanoparticles resulted in a flexural strength decrease of up to 2% at 1% (*w*/*w*) and up to 12% at 5% (*w*/*w*). The flexural strength increased in CR2 clay up to 6% at 1% (*w*/*w*) and up to 3% at 5% (*w*/*w*) with the addition of Fe_3_O_4_ nanoparticles. Notably, the flexural strength results of the test tiles surpassed the standard value (800 N) established by ISO 13006.

Figure 7e represents that the 1% and 5% (*w*/*w*) mixtures containing nanoparticles showed an increase in the temperature gradient compared to the 0% (*w*/*w*) mixture. In the case of CR1 clay, the temperature gradient increased up to 3% at 1% (*w*/*w*) and up to 2% at 5% (*w*/*w*) when Fe_3_O_4_ nanoparticles were added. For CR2 clay, the temperature gradient increased up to 9% at 1% (*w*/*w*) and up to 2% at 5% (*w*/*w*) with the addition of Fe_3_O_4_ nanoparticles. Nanoparticle additions increased the temperature gradient of clays due to their high specific surface area and reduced size, altering the clay structure and increasing surface reactivity and thermal energy release during firing.

The results of this study indicate that Fe_3_O_4_ nanoparticles added at 1% (*w*/*w*) are the most effective additive for improving both flexural strength and temperature gradient in CR2 and TF clay. The improvement of the apparent thermal conductivity of TF clay test tiles has increased up to 32%. This improvement in thermal conductivity results in significant energy savings that can be used to optimize drying and firing processes in tile manufacturing. The higher apparent thermal conductivity of the tiles allows faster drying times and lower firing temperatures, which reduces the overall energy consumption of the process. In addition, the use of magnetite nanoparticles at 1% (*w*/*w*) is effective in enhancing the flexural strength of test tiles clay (up to 16%), enabling a reduction in thickness without compromising their mechanical properties. By reducing the thickness of tiles, their mass is decreased, resulting in lower energy usage during manufacturing. This leads to significant energy savings in the extraction, transportation, processing, drying, and firing processes. Thus, the incorporation of magnetite nanoparticles improves the sustainability of tile production while also reducing energy and material consumption.

### 3.4. Microstructural and Morphological Analysis TF Test Tile with Fe_3_O_4_ Nanoparticles Addition

**Figure 8:**
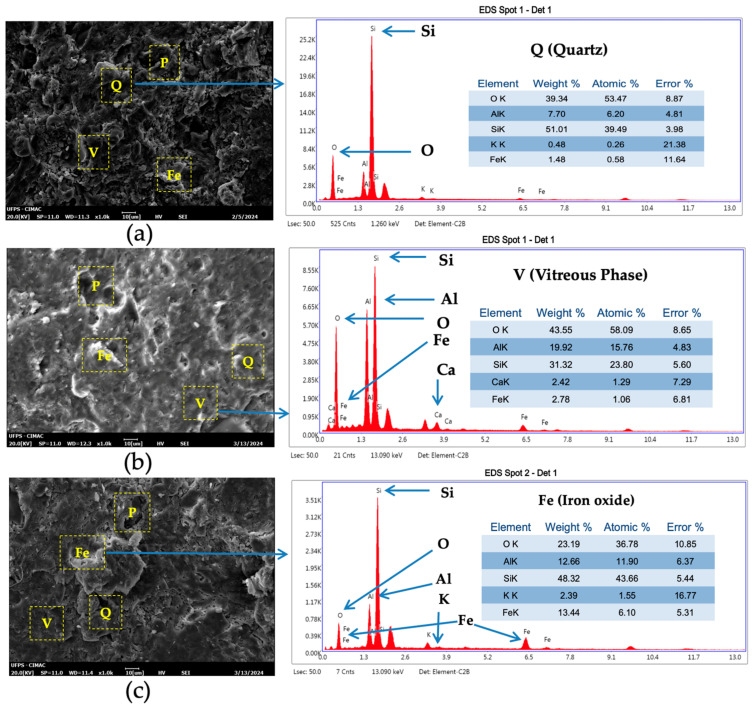
SEM images corresponding to test tiles of TF clays fired at 1120 °C with (**a**) 0%, (**b**) 1% and (**c**) 5% (*w*/*w*) Fe_3_O_4_ nanoparticle additions.

Figure 8 displays a SEM micrograph illustrating at 1000× magnification the characteristic structure of TF fired test tiles. Within the microstructure, dark-grey areas denoted as Q represents quartz grains, while black regions labeled as P indicate irregular-shaped pores dispersed amidst a continuous glassy phase, represented by light-grey zones labeled as V [42,80]. Additionally, the EDS shows presence of iron oxide, depicted by bright zones marked as Fe, attributed to the Fe_2_O_3_ content in the TF clay (see Table 1) and the transformation of the magnetite Fe_3_O_4_ [81].

**Figure 9:**
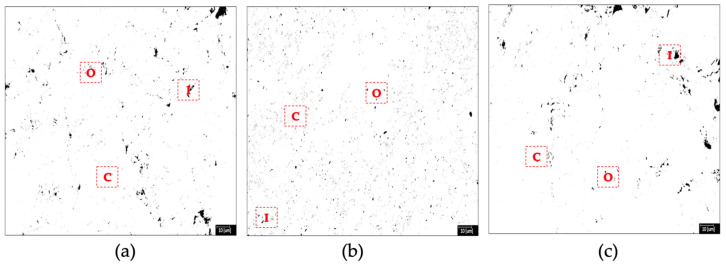
SEM images were used to calculate the total porosity of test tiles with TF clay. (**a**) 0%, (**b**) 1% and (**c**) 5% of Fe_3_O_4_ (*w*/*w*) nanoparticles addition.

The total porosity percentage and sample size of the TF clay and magnetite nanoparticles were carried out using ImageJ (version 1.54) image processing software [82,83,84]. In their morphology (Figure 9), all test tiles, whether control or with nanoparticles addition, showed different types of porosity (black zone), open (O), closed (C) and interparticle (I) [85]. Open porosity, characterized by interconnected and irregularly shaped pores of small sizes is present. Additionally, closed porosity is represented by spherical isolated pores of large sizes, while interparticle porosity is associated with irregularly shaped pores situated at crystalline grain boundaries [86,87]. This final porosity has its origin in the different coefficients of thermal expansion between the glassy and crystalline phases. Table 4 shows test tiles with 1% of nanoparticles addition display a reduction in pore formation (1.246%), hinting at potential improvements in sintering behavior [88]. This decrease in porosity is related to low water absorption and high flexural strength of TF clay with magnetite NPs addition.

### 3.5. Life Cycle Assessment of Test Tile Clay Manufacturing

This life cycle analysis was conducted to assess the potential environmental impacts of the ceramic tile production processes using CR1, CR2 and TF clays. Specifically, the analysis evaluated the impacts of producing ceramic tiles without nanoparticles addition and magnetite (Fe_3_O_4_) nanoparticles addition at 0% and 1% (*w*/*w*).

These results do not evaluate the toxicity and carcinogenicity of the test tiles by the nanoparticles addition. Instead, they focus on the environmental impacts generated from raw material extraction to final product manufacturing (cradle to gate), encompassing raw materials and energy consumption. These environmental impacts are reflected in the life cycle assessment indicators of each process.

**Figure 10:**
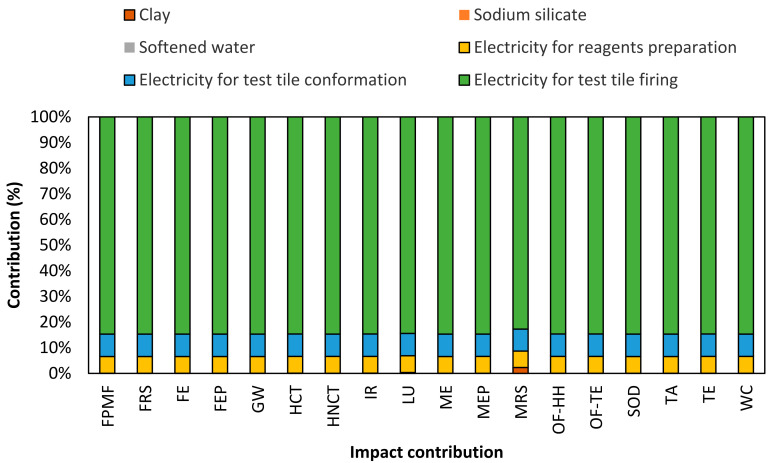
Normalized contribution per impact category of clay without nanoparticles addition (0% *w*/*w*). The abbreviation definitions of the impact categories are listed in Section 2.9.

Figure 10 presents information on the life cycle assessment of the manufacture of test clay tiles without nanoparticle addition (0% *w*/*w*). LCIA results showed that the electricity contributes up to 100% to the total of all impact categories. This result can be explained by high consumption of electrical energy in all processes, especially the firing process (85%), due to the high temperature (1120 °C) and longtime exposure required to achieve the end properties [9,89,90]. Findings indicated that the greatest negative impacts were terrestrial ecotoxicity, global warming and non-carcinogenic human toxicity, with contribution values up to 61%, 16% and 14%, respectively.

**Figure 11:**
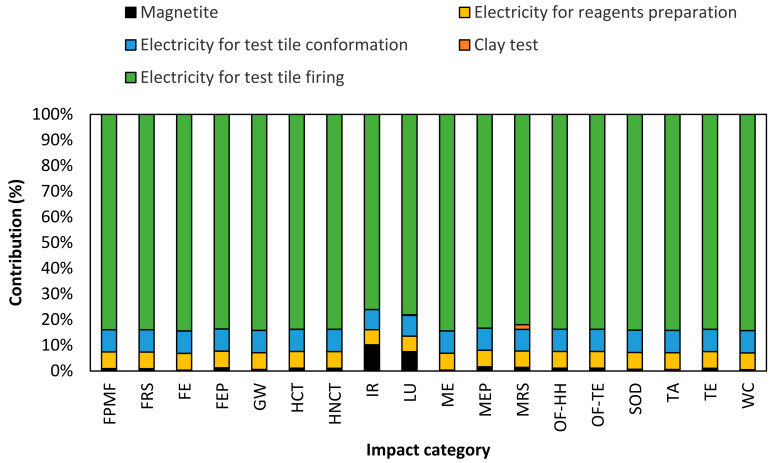
Normalized contribution per impact category of test tiles clay manufacturing with magnetite nanoparticles addition at 1% (*w*/*w*). The abbreviation definitions of the impact categories are listed in Section 2.9.

Figure 11 shows the test tile samples with addition of magnetite (Fe_3_O_4_) nanoparticles at 1% (*w*/*w*). The LCIA results showed that electricity consumption and magnetite nanoparticles contribute up to 98% and 2%, respectively, in all impact categories. On the other hand, results indicate that the clay does not contribute significantly in the LCA; on average, the contribution was 0.2% in the impact categories. Consequently, negative impacts were observed on the environment, such as terrestrial ecotoxicity, global warming, and human non-carcinogenic toxicity, with contribution values of 99%, 98%, and 95%, respectively. These results highlight the importance of finding ways to reduce electricity consumption and its associated impacts on the environment in both ceramic processes and magnetite synthesis.

Table 5 compiles the outcomes of a life cycle environmental impact assessment (LCIA) conducted on ceramic test tiles incorporating magnetite nanoparticles in two different scenarios. Scenario 1 (without nanoparticles addition—0% *w*/*w*) is the baseline case without energy and mass savings, while scenario 2 (with magnetite nanoparticles addition at 1% *w*/*w*) includes a 48% energy saving and a 16% mass reduction. This improvement is attributed to a substantial 32% increase in the apparent thermal conductivity and a 16% boost in flexural strength facilitated by the addition of magnetite nanoparticles. Notably, scenario 2 achieved a significant decrease in several environmental impact categories, including a 20% reduction in global warming potential, a 19% reduction in non-carcinogenic toxicity to humans, and a 19% reduction in terrestrial ecotoxicity compared to scenario 1. This reduction was primarily due to the reduced energy consumption and material inputs achieved by improving the thermal conductivity and flexural strength of the tiles. In summary, our findings underscore the potential magnetite nanoparticles integration to mitigate the environmental impacts associated with ceramic tile manufacturing.

## 4. Discussion

Table 6 shows that studies and patents have been conducted on a range of nanoparticles for various ceramic tile applications. These involve surface functionalization, including coatings, for specialized applications, such as TiO_2_ for tile self-cleaning, SiO_2_-TiO_2_ for photocatalysis, and Ag-TiO_2_ and Cu for antibacterial activity. The enhancement of specific properties, such as flexural strength and compressive strength in the ceramic body, has been achieved through the incorporation of Al_2_O_3_, Fe_2_O_3_, PbO and SiO_2_ nanoparticles. In comparison to previous studies, our research focuses on integration of Ag_2_O, CuFe_2_O_4_, Fe_3_O_4_, and SiO_2_ nanoparticles into ceramic body tiles to enhance their mechanical and thermal properties, specifically their flexural strength and apparent thermal conductivity. This approach reduces energy and material consumption, and mitigates environmental impacts in the life cycle assessment of ceramic manufacturing processes.

The results indicate that the addition of magnetite (Fe_3_O_4_) nanoparticles at 1% (*w*/*w*) had a positive effect on both flexural strength and apparent thermal conductivity compared to tiles at 0% (*w*/*w*) nanoparticles addition. The apparent thermal conductivity related to the temperature gradient was higher in clays with magnetite (Fe_3_O_4_) nanoparticles addition: CR1 (+3%), CR2 (+9%) and TF (+32%), at 1% (*w*/*w*) compared to nanoparticles addition at 0% (*w*/*w*). Studies, such as the one conducted by Wang et al. [91], have demonstrated that certain elements inherent in clays (notably iron (Fe)), significantly influence their thermal, physical, and mechanical properties, including thermal conductivity [66,92,93]. Notably, CR2 and TF clays with 1% (*w*/*w*) magnetite (Fe_3_O_4_) nanoparticles showed an increase up to 6% and 16%, respectively, in flexural strength. Therefore, the thickness or weight of ceramic tiles can be decreased up to 16% in TF clay without degrading their flexural strength properties [9]. According to Türkmen et al. [54], a 7% reduction in thickness results in a decrease in energy consumption for the spray dryer by 4% and for firing by 9%.

Some previous studies, such as Bazzocchi et al. [19], investigated thinning porcelain stoneware tiles without compromising flexural strength, finding minimal impact from the glass fiber network. Our research achieved a notable increase in flexural strength while lowering energy consumption at 1120 °C firing temperature, enhancing sustainability in ceramic tile production. In contrast, Da Silva et al. [20] achieved a 42% flexural strength increase at 1220 °C, compared to our study, an increase up to 87% was achieved at 1120 °C, demonstrating the reduction in environmental impacts. Additionally, examined reinforced fiberglass to maintain mechanical properties, achieving a 42% increase in flexural strength, emphasizing diverse strategies for ceramic tile enhancement without thickness increase.

Chen et al. [40] explored the effect of incorporating nano-SiO_2_ particles (mean particle size of 10 ± 5 nm) into ceramic tiles. Their study, incorporating nano-SiO_2_ fractions ranging from 0 to 3% by weight, revealed a reduction in water absorption to less than 12% at 1100 °C. Additionally, an increase in flexural strength by 0.5–2.5 MPa was observed after the addition of 2% nano-SiO_2_. In the study by Mahmoud et al. [41], porcelain ceramic tiles were doped with lead oxide (PbO) nanoparticles, which found that as their content in the ceramic body increased from 0.0 to 10.0% wt., the modulus of rupture increased from 1.753 ± 0.07 to 2.674 ± 0.11 MPa, respectively. This improvement was attributed to the partial substitution of the clay mixture in the new ceramic nanocomposites, promoting liquid-phase sintering and higher density in the tiles. A similar trend was noted in the study by De La Garza et al. [42], where the influence of alumina and silica nanoparticles at 1% in porcelain stoneware improved compressive strength (65.27 and 55.37 MPa), confirming the role of nanoparticles in promoting denser microstructures during the sintering process. Additionally, Nawaukkaratharnant et al. [43] reported a notable flexural strength increase with rising firing temperatures, particularly at 3% and 5% wt. Fe_2_O_3_, additions, exceeding 30 MPa after firing above 1150 °C. Conversely, the introduction of 7% wt. Fe_2_O_3_ negatively impacted flexural strength due to the altered porosity and glassy phase influenced by the Fe_2_O_3_ content. The patent by Fix-Fierro et al. [44], conditioned a family of additives such as sodium, potassium, boron, silicon, zinc, and calcium oxides, among others, that form nanometric structures and allow one to obtain a powder capable to decrease the sintering temperature of a ceramic red body from 1180 °C to 1050 °C (up to 13%) due to high fluxing power. Our study confirms that the increase in apparent thermal conductivity is attributed to the higher fluxing power resulting from the addition of 1% magnetite nanoparticles. This effect is enhanced by reducing porosity, as illustrated in Figure 9, allowing for a reduction from 1120 °C to 975 °C in sintering temperature and, consequently, in energy consumption.

The Life Cycle Assessment (LCA) results highlighted the substantial impact of energy consumption during the drying and firing processes of ceramic tiles with magnetite nanoparticle additions at 0% and 1% (*w*/*w*). Electricity consumption dominated in all categories, particularly affecting terrestrial ecotoxicity, global warming, and human non-carcinogenic toxicity, contributing 99%, 98%, and 95%, respectively. This result was due to the high temperature (1120 °C) firing process and long exposure [9,89,90,94,95]. The comparison with the other studies presents some difficulties due to different methods and software used, and is a challenge for the LCA community [96].

In comparison to previous research, our study stands out for its innovative approach to reducing energy consumption and improving the properties of ceramic tiles. For instance, Türkmen et al. [54] demonstrated significant decreases in energy consumption during drying and firing, as well as environmental impacts, by reducing tile thickness. Similarly, Blundo et al. [97] found that porous ceramic tiles exhibited considerable environmental impacts, particularly on human health. In contrast, our study evaluated the use of magnetite nanoparticles in ceramic tiles, achieving an up to 48% reduction in energy consumption and improvements in physical properties. Specifically, we observed a 20% decrease in global warming potential and a 19% reduction in non-carcinogenic toxicity and terrestrial ecotoxicity compared to the baseline scenario without nanoparticles. These findings underscore the potential of magnetite (Fe_3_O_4_) nanoparticles at 1% (*w*/*w*) to mitigate environmental impacts in ceramic tile production.

## 5. Conclusions

The results of our study open a promising horizon for the future of sustainable ceramic tile production. These findings are not only highly relevant to the ceramic industry, but also extend to the scientific community, emphasizing the pivotal role of nanotechnology in enhancing tile properties and energy efficiency in production. Furthermore, they provide an inspiring vision for the next generation of research and developments in this field, underscoring the significance of innovation in the evolution of ceramic tile production towards new sustainability standards. These conclusions reflect a commitment to a cleaner and more efficient future, where science and the ceramic industry come together to drive progress towards a greener and more sustainable world.

The objective of many companies in the world and especially in the ceramic sector is to reduce their impact on the environment. The ceramic sector is one of the highest in terms of energy and materials consumption. The new technologies and materials have the potential to help the ceramic tiles sector achieve a substantive reduction in this area. This study demonstrated that this can be achieved by adding nanoparticles to the ceramic tile body. Results showed that the addition of magnetite nanoparticles at 1% (*w*/*w*) in TF clay improved the apparent thermal conductivity up to 32% and flexural strength up to 16%. In the future, these improvements in the apparent thermal conductivity will allow further exploration and creation of new kinds of products and new uses for them. The nanoparticle additions represent an alternative for reducing the thickness or decreasing the weight of the ceramic tiles without affecting one of the most important mechanical properties, flexural strength.

Furthermore, the LCA to produce test tiles with the addition of nanoparticles was evaluated, and it was demonstrated that an improvement of the apparent thermal conductivity and flexural strength can decrease the energy and materials impacts of the process on the LCA. In the sample tiles manufacturing process, results showed that the electrical energy contributes up to 100% to the total impact on the categories, which most affect terrestrial ecotoxicity, global warming, and human non-carcinogenic toxicity. The findings indicated that Scenario 2 with addition of magnetite nanoparticles at 1% (*w*/*w*) significantly reduced the environmental impacts, up to 20% in global warming potential, up to 19% in non-carcinogenic toxicity and up to 19% in terrestrial ecotoxicity compared to Scenario 1 at 0% (*w*/*w*).

## Figures and Tables

**Table 1:** Particle sizes and chemical compositions of clays CR1, CR2 and TF by X-ray fluorescence (XRF).

	Clays		
	CR	CR2	TF
Particle size Oxide Compound (wt.%)	12.7 mm > 22.0% > 7.9 mm		
	7.9 mm > 4.6% > 6.4 mm		
	6.4 mm > 7.8% > 4.0 mm		
	4.0 mm > 33.6%> 1.0 mm		
	1.0 mm < 32.0%		
SiO_2_	68.05	65.47	63.84
Al_2_O_3_	17.73	19.33	20.76
CaO	0.23	0.23	0.39
MgO	0.64	0.68	0.70
Na_2_O	0.25	0.20	0.19
K_2_O	1.80	1.90	2.05
Fe_2_O_3_	4.33	4.41	4.05
TiO_2_	0.94	0.97	0.88
SO_3_	0.04	0.03	0.04
V_2_O_5_	0.03	0.04	0.05
P_2_O_5_	0.09	0.10	0.09
ZrO_2_	0.05	0.04	0.04
SrO	0.04	0.04	0.04
Mn0	0.04	0.04	0.02
ZnO	0.03	0.03	0.04
Cr_2_O_3_	0.02	0.02	0.02
LOI	5.67	6.49	6.79

**Table 2:** Inventory report of manufacturing process for test samples.

Method		Inventory	Amount	Unit
Laboratory-scale process	Reagents preparation	Inputs		
		Clay	500	g
		Water	269	g
		Sodium silicate	4	g
		Magnetite	5	g
		Electricity (Weighing machine)	0.0001	kWh
		Electricity (Grinder Speedy)	0.026	kWh
		Electricity (Drying oven)	1.840	kWh
	Powder conformation	Water	25.20	g
		Electricity (Grinder ct-12061)	0.01	kWh
	Test tile conformation	Electricity (Manual press)	0.24	kWh
		Electricity (Drying oven)	2.484	kWh
	Test tile firing	Electricity (Oven)	24.29	kWh
		Outputs		
		Test tile	420	g

**Table 3:** Mineralogical analysis (in wt.%) obtained by XRD determination using CR1, CR2 and TF clays.

Mineral Phases wt.%	Clays		
	CR1	CR2	TF
Quartz	42	41	36
Kaolinite 1A	24	27	45
Microcline	2	2	2
Anatase	<1.0	2	1
Nacrita-2M2	6	4	3
Muscovite-2M1, ferric	23	23	11
Albite	2	1	1
Hematite	<1.0%	<1.0%	<1.0%

**Table 4:** Total porosity and average size of TF clay.

Clay + Fe_3_O_4_ NPs	Total Porosity %	Average Size µm
TF—0%	1.420	0.220
TF—1%	1.246	0.068
TF—5%	1.384	0.304

**Table 5:** Life cycle impact assessment (LCIA). Scenario 1 (clays at 0% nanoparticles addition). Scenario 2 (clays with magnetite nanoparticles at 1% *w*/*w*) includes energy saving and mass reduction.

Impact Category	Abbreviation	Reference Unit	Clays at 0% (*w*/*w*) Fe_3_O_4_ NPs	Clays at 1%(*w*/*w*) Fe_3_O_4_ NPs
Fine particulate matter formation	FPMF	kg PM2.5 eq	0.30	0.24
Fossil resource scarcity	FRS	kg oil eq	39.30	31.57
Freshwater ecotoxicity	FE	kg 1,4-DCB	33.31	26.54
Freshwater eutrophication	FEP	kg P eq	0.04	0.04
Global warming	GW	kg CO_2_ eq	216.51	173.20
Human carcinogenic toxicity	HCT	kg 1,4-DCB	10.38	8.37
Human non-carcinogenic toxicity	HNCT	kg 1,4-DCB	187.36	150.88
Ionizing radiation	IR	kBq Co-60 eq	0.95	0.87
Land use	LU	m2a crop eq	0.39	0.35
Marine ecotoxicity	ME	kg 1,4-DCB	40.74	32.47
Marine eutrophication	MEP	kg N eq	0.00	0.00
Mineral resource scarcity	MRS	kg Cu eq	0.56	0.45
Ozone formation, Human health	OF-HH	kg NOx eq	0.32	0.26
Ozone formation, Terrestrial ecosystems	OF-TE	kg NOx eq	0.33	0.26
Stratospheric ozone depletion	SOD	kg CFC11 eq	0.00	0.00
Terrestrial acidification	TA	kg SO_2_ eq	0.90	0.72
Terrestrial ecotoxicity	TE	kg 1,4-DCB	849.10	683.75
Water consumption	WC	m^3^	4.21	3.363

**Table 6:** Studies and patents on application of nanoparticles in ceramic tiles.

Type of Nanoparticles	Applications		Particle Size (nm)	*w*/*w* %	Results	Author
	Body Tile	Coating				
TiO_2_		Self-cleaning ceramic tiles	9–67	5	Self-cleaning ceramic tiles	Da Silva et al. [28]
SiO_2_-TiO_2_		Self-cleaning ceramic tiles	5	3.4	Self-cleaning coatings and photocatalytic ceramic	Ferreira-Neto et al. [29]
Ag-TiO_2_		Photoactive tiles	110–130	8	Oxidate inactivation of SARS-CoV-2	Djellabi et al. [30]
Ag-TiO_2_		Photocatalytic antibacterial tile	110–130	1, 4 and 8	Photocatalytic ability	Bianchi et al. [31]
ZnO		Glazed ceramic tiles.	38	10	Photocatalytic activity	Guzman-Carrillo et al. [32]
SnO_2_, ZnO, Sb_2_O_3_		Glazed ceramic countertop.	50–100	0.5–6	Excellent antibacterial and antistatic properties	Ke et al. [33]
SiO_2_, Ge		Glazed porcelain tiles	Not available	2–3	Odorless and free toxics	Liu et al. [34]
Cu(NO_3_)_2_		Anti-microbial coatings	Not available	0.03 to 3	Stable at temperatures up to at 1350 °C	Darragh et al. [35]
Au, Ag, Cu, Pl, Ti		Inkjet ink for a ceramic substrate	5.0–90	0.05 to 0.8	Better fixed on a ceramic substrate	Hiromichi et al. [36]
Mg		Photocatalytic antibacterial tile	50	0.5–5	Level 0 anti-mildew	Wang et al. [37]
SiO_2_, TiO_2_		Photocatalytic antibacterial tile	10	50–90	Excellent photocatalytic activity even after 1000 °C	Ferreira-Neto et al. [38]
Cu		Glazed ceramic tiles.	30–90	3	Antibacterial efficiency of 99.9%	Kim et al. [39]
SiO_2_	Pottery and porcelain clays		10	0–3	Increase in flexural strength 0.5–2.5 Mpa	Chen et al. [40]
PbO	Ceramic tiles for γ-ray shielding		29.64	0–10	Increase in modulus of rupture 2.674 Mpa	Mahmoud et al. [41]
Al_2_O_3_ and SiO_2_	Porcelain stoneware		20–30	0.25–1	Compressive strength 55.37 and 65.27 Mpa	De la Garza et al. [42]
Fe_2_O_3_	Red stoneware		Not available	3–7	Flexural strength 30 Mpa	Nawaukkaratharnant et al. [43]
Si, Ca, Na, Mg, B, Zn, Al, P	Red and porcelain stoneware		50–500	0.2–5	Sintering temperature reduction up to 13%	Fix-Fierro et al. [44]
AgO_2_	Red stoneware		15	1–5	Increase water absorption up to 26%, decrease flexural strength up to 13% and increase of temperature gradient up to 24%.	This study
CuFe_2_O_4_	Red stoneware		100	1–5	Decrease water absorption up to 33%, increase flexural strength up to 9% and increase temperature gradient up to 18%.	This study
Fe_3_O_4_	Red stoneware		88.59	1–5	Decrease water absorption up to 40%, increase flexural strength up to 16% and increase temperature gradient up to 32%.	This study
SiO_2_	Red stoneware		20	1–5	Increase water absorption up to 28%, decrease flexural strength up to 16% and increase of temperature gradient up to 21%.	This study

## Data Availability

The data and contributions presented in the study are included in the article. Further inquiries can be directed to the corresponding author.
